# NO_2_ Sensing with SWCNT Decorated by Nanoparticles in Temperature Pulsed Mode: Modeling and Characterization

**DOI:** 10.3390/s20174729

**Published:** 2020-08-21

**Authors:** Enza Panzardi, Anna Lo Grasso, Valerio Vignoli, Marco Mugnaini, Pietro Lupetti, Ada Fort

**Affiliations:** 1 Department of Information Engineering and Mathematical Sciences, University of Siena; via Roma 56, 53100 Siena, Italy; panzardi@diism.unisi.it (E.P.); alograsso@diism.unisi.it (A.L.G.); valerio.vignoli@unisi.it (V.V.); marco.mugnaini@unisi.it (M.M.); 2 Department of Life Sciences, University of Siena; via Aldo Moro 2, 53100 Siena, Italy; pietro.lupetti@unisi.it

**Keywords:** SWCNT (Single-wall carbon nanotube) NO_2_ sensor, TiO_2_ nanoparticles, Au nanoparticles, dynamic models

## Abstract

In this paper, NO_2_ sensing by means of single-wall carbon nanotubes (SWCNT) networks, decorated with nanoparticles of TiO_2_ and Au, is proposed. In particular, it is shown that the performance of these materials can be enhanced using pulsed temperature mode. This sensing strategy effectiveness is theoretically and experimentally assessed. In this paper, in fact, a dynamic model for conductive gas sensors formed by networks of nanowires, considering the junctions between different wires as the main contribution to sensor conductance, and in the presence of the target gas, is presented and validated. The model accounts for variable temperature and gas concentration and sheds some light on the mechanisms leading to the sensor response improvement related to temperature pulsed working mode. It is also shown how the addition of a different material can be modeled through different surface adsorption kinetics.

## 1. Introduction

The suitability of single-wall carbon nanotubes (SWCNT)-based materials as sensing layers for toxic gas detection has been widely investigated in the last years [1,2,3,4,5], and recent researches have shown that they can be successfully employed to realize conductive gas sensors with promising performance. The achieved results point out the potentialities of these materials, but a large effort is still needed to reach the exhaustive knowledge of their gas sensing properties [6], which is needed to enable the evolution of commercial solutions employing SWCNT-based sensors.

The behavior of SWCNT-based gas sensors is very complex and still an open research subject; therefore, at present, a reliable SWCNT sensor input-output model able to predict the gas sensor behavior is not available. As a consequence, gas sensor-based measurement systems are designed and tuned exploiting experimental characterization. In this context, it is clear how modeling is of the utmost utility under many points of view. First of all, the availability of a simulation tool, able to predict with sufficient accuracy the sensor behavior, enables to replace experimental tuning with simulations, and can significantly speed-up sensor-based system development and guarantee better performance. Moreover, and perhaps more important, the comparison of the model outputs with experimental data can greatly help to understand the behavior of SWCNT-based sensor, because it explores the relevance of the different mechanisms involved in sensing, to validate some commonly accepted assumptions, or to assess their validity ranges.

In general, conductive gas sensor models have to incorporate all the phenomena that contribute to the sensing mechanism, which are very complex and comprise chemical solid-gas reactions and physical phenomena related to electronic conduction. Especially the first ones are very difficult to be observed and often remain the subject of hypotheses difficult to assess with independent measurements.

In detail, a sensor model has to describe, at first, the interaction of the target gases with the sensing material, due to the surface chemical reactions.

Secondly, the model must account for the relationship of the resistance variation with the quantity of adsorbed gas. Different mechanisms can occur in different film microstructures [7,8,9,10,11,12]. In particular, for many semiconducting materials the electronic conduction, and hence, the resistance value, is mainly determined by the surface chemical reactions, that are responsible for the creation of charged species trapped on the surface. The presence of adsorbates affects the resistance in a way that depends on the film micro-structure. For SWCNT networks, for instance, it appears possible that the most relevant phenomenon for the sensor response is the influence of adsorbed and charged species on the inter-tube contact barriers within CNT fibers. This assumption is also supported by the results obtained by the authors for Metal oxides nanowires bundles in References [13,14,15], however, the bulk electrical and thermal conductivities of CNT ensembles were found much lower than those of the individual nanotubes. Firstly, inter-tube contacts within CNT fibers (CNTFs) induce large interfacial resistances, which are verified to be the main barriers for electron and phonon transport [16,17].

In this paper, which is an in-depth study and is complementary to what the authors already published in Reference [3], the response of SWCNT networks is modeled following an approach similar to the one presented by the authors in References [13,18,19,20] which leads to the development of a gray-box model that, based on the physical and chemical description of the chemisorption reactions, provides a compact description of the sensor behavior. The model explains the sensor dynamics by means of direct adsorption processes of NO_2_ and oxygen on the CNT sidewalls. This approach can also be applied to large-grained thick film sensors or to nanowire (quasi 1-D) bundles and takes into account the sensor operation under dynamic thermal and/or chemical conditions.

The developed model is used to explore the possibility of using SWCNT networks decorated with TiO_2_ and Au nanoparticles [3] for the detection of NO_2_ using pulsed temperature profiles [21] with the aim of obtaining optimized sensor response and of reducing the power consumption. In particular, the effect of operating the sensors by means of variable working temperature (consisting of a pulse train (rectangular wave) that rapidly switches between a high and low temperature) was investigated.

The structure of the paper is the following. In Section 2, the sensors used in this study are described, and results concerning the sensing material characterization are presented. In Section 3, a novel gray-box model of the dynamic behavior of SWCNT networks-based NO_2_ sensor is derived starting from a theoretical chemical/physical analysis. In Section 3, the model calibration, i.e., the parameter estimation procedure is described, whereas Section 4 presents simulations obtained with the as-calibrated model. The simulation study is aimed at choosing an optimized pulsed temperature profile, which is then used to obtain the experimental results presented and discussed in Section 5. Finally, in Section 6, the conclusions are drawn.

## 2. Sensor Preparation and Material Characterization

### 2.1. Materials

The SWCNTs were obtained from Sigma Aldrich (Merck KGaA, Darmstadt, Germany) in powder form, at ≥95% purity; they present a semiconducting tube content >95%, (6,5) chirality and a diameter in the range of 0.7–0.9 nm. The gold nanoparticles were purchased in suspended solution from Sigma Aldrich the nanoparticles diameter is approximately 5 nm with a concentration of about 5.5 × 10^13^ particles/mL in a phosphate-buffered saline (PBS) base solution (0.1 mM). The TiO_2_ nanoparticles were obtained from Italvernici (Italvernici Srl, Ponsacco, Italy) in a water suspended solution with a concentration of 32 × 10^−3^ mol/L and a nanoparticle diameter approximately in the range 25–55 nm.

### 2.2. Sensors Preparation

The sensing films, as well as the sensors’ structure, used in this work were realized according to the procedure described in detail in Reference [3]. For completeness and clarity, a brief summary is reported here.

The SWCNT-based solution was obtained by dispersing the SWCNT in a solution of surfactant sodium dodecyl-benzenesulfonate and water (0.01 wt.%). Afterwards, magnetic mixing and sonification procedures were performed in order to ensure the dispersion of the material in the liquid phase.

The gas sensor prototypes were realized in screen printing technology using an alumina substrate. One side of the device hosts the sensor heater, whereas the other one hosts a Pt-based temperature sensor and two silver interdigitated electrodes.

**Figure 1:**
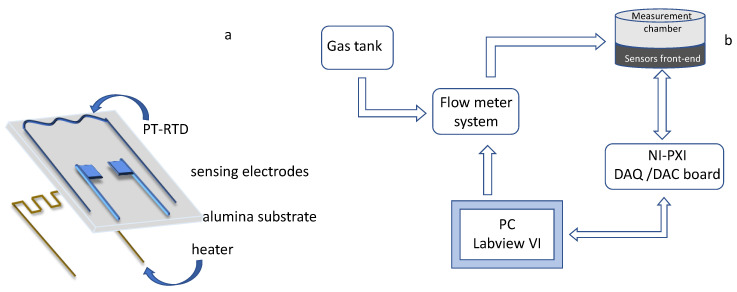
Schematic representation of the sensors’ structure (**a**) and of the gas measurement and characterization system (**b**).

In Figure 1a, a schematic representation of the sensor structure is reported. The sensing film is realized by depositing 1 μL of the SWCNT-based solution between the silver electrodes by drop-casting. The prepared sample is then heated at 400 °C for 24 h, when it is ready for the decoration procedure. The Au and TiO_2_ nanoparticles are drop-casted on the obtained SWCNT films through depositions of 1 μL of the decorating suspended solutions. The sensor samples are then dried at room temperature and subsequently heated at 400 °C for four hours. The decorated sensor prototypes considered in this work are of two types, the first one is realized by three subsequent depositions of Au nanoparticles (corresponding approximately to 59 ng of deposited mass), and the second one is realized by two subsequent depositions of TiO_2_ nanoparticles (approximately 5 μg of deposited mass).

The pristine sensors have been cleaned with ultrapure-water and then heated at 400 °C for two hours in order to remove residual impurities, before the sensing film deposition procedure. Eight sensors samples were prepared and tested. Four of them were decorated by subsequent TiO_2_ depositions, whereas four were decorated by subsequent Au nanoparticle depositions.

Details about the measurement and characterization system used to acquire the sensor responses, as well as the used sensor structure, can be found in References [22,23,24], and a schematic representation is reported in Figure 1. It is worth to notice that a deeper investigation concerning the performance of the proposed sensing devices, in terms of response time, cross-sensitivity to different target gases and humidity levels, was presented and discussed by the authors in Reference [3]. The system remotely manages and controls the working conditions in the gas measurement chamber in terms of gas concentration and species, keeping the total gas flow constant during measurements thanks to a digitally controlled gas flow meter system (BronkHorst F-201C). Moreover, it is possible to vary and control, with a feedback strategy exploiting the embedded Pt temperature sensor (Pt-resistive temperature detector, Pt-RTD, in Figure 1) the working temperature of each of the tested sensor with an accuracy lower than 3 °C in the range [120 °C–400 °C]. The gas measurement chamber is in steel and can host up to 8 sensors, which can be tested at the same time. In this work, the total gas flow was kept constant at 200 mL/min, and the measurement profile (temperature and gas concentration in time) varied according to the need. The repeatability obtained with the same sensors is about 10% of the response value, and the estimated reproducibility is about 20% of the response value with this preparation route.

### 2.3. Material Characterization

The chemical composition and morphological characteristics of the realized sensing films were analyzed by Scanning and by Transmission electron microscopy using a Quanta 400 (FEI) emission scanning electron microscope (SEM) and a Tecnai G2 Spirit (FEI) transmission electron microscopy (TEM) operating at 20.0 kV and 120 kV, respectively.

**Figure 2:**
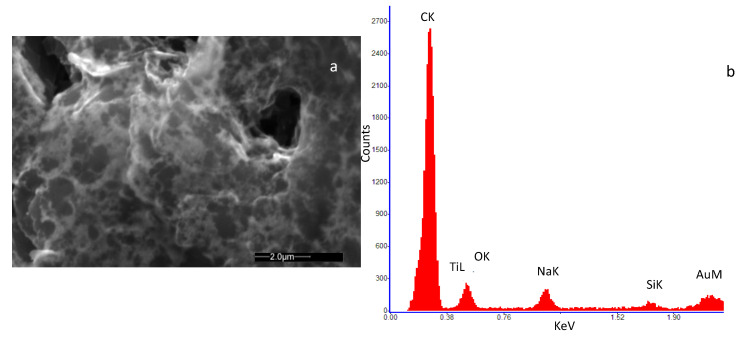
High-resolution field emission scanning electron microscopy (SEM) image of the deposited film of SWCNTs decorated by Au nanoparticles. The microscopic investigation was performed with a Quanta 400 (FEI) emission scanning electron microscope, operated at 20.0 kV. (**a**) view of the deposited film; (**b**) result of the Energy Dispersive Spectroscopy (EDS) on the analyzed sample.

**Figure 3:**
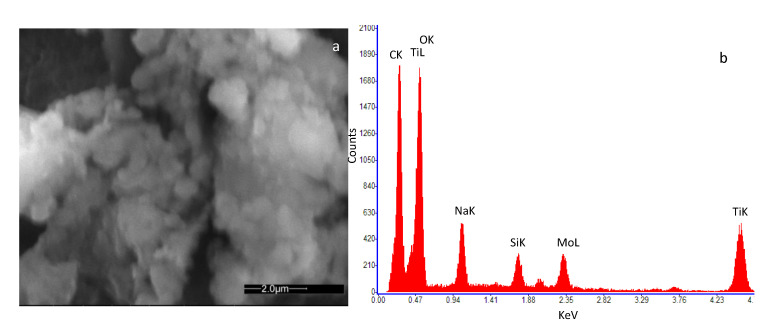
High-resolution field emission scanning electron microscopy (SEM) image of the deposited film of SWCNTs decorated by TiO2 nanoparticles. The microscopic investigation was performed with a Quanta 400 (FEI) emission scanning electron microscope, operated at 20.0 kV. (**a**) view of the deposited film; (**b**) result of the Energy Dispersive Spectroscopy (EDS) on the analyzed sample.

Figure 2a and Figure 3a show an SEM image of the SWCNT sample decorated with Au and TiO_2_ nanoparticles, respectively. In Figure 2b the Energy Dispersive Spectroscopy analysis (EDS) on the SWCNT-Au sample is reported, indicating the presence of carbon as the main element together with a small amount of Au, confirming the decoration of the base material. Traces of Ti, Na and Si were also detected at impurity levels, which may be originated during the material and sensor preparation procedure (solution preparation and drying procedure). In Figure 3b the EDS analysis of the SWCNT-TiO_2_ sample is shown. Once again, the main detected element is carbon followed in concentration by oxygen and titanium, as to confirm the SWCNT decoration. For this sample, also, traces of Na and Si were detected at impurity levels.

**Figure 4:**
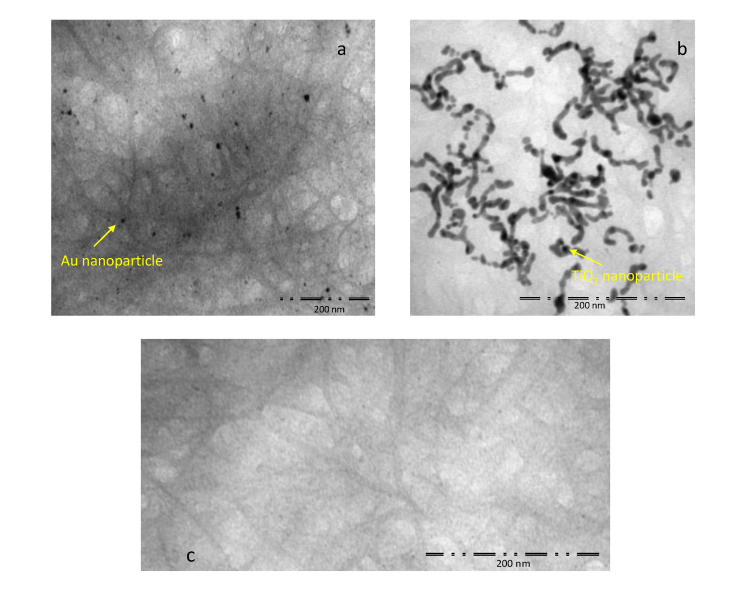
High-resolution Transmission Electron Microscopy (TEM) images of the decorated samples: (**a**) SWCNT-Au nanoparticles, Scale bar = 200 nm; (**b**) SWCNT-TiO_2_ nanoparticles, Scale bar = 200 nm; (**c**) SWCNT, Scale bar = 200 nm; the investigation was performed using a Tecnai G2 Spirit (FEI) operated at 120 kV.

TEM images of the investigated nanostructures are presented in Figure 4. The decoration of TiO_2_ and Au nanoparticles on the CNT sidewalls is visible in Figure 4a,b, respectively. The comparison with the image of a pure SWCNT sample (Figure 4c) confirms the presence of the Au and TiO_2_ nanoparticles in the SWCNT network, realizing the sensor decoration.

## 3. Model Derivation.

SWCNTs used for gas sensing applications are semiconductors with a band-gap depending on their molecular structure [25,26,27] (as a general rule of thumb, the observed bandgaps are roughly proportional to the reciprocal of the tube radius) in which unintentional defects act as acceptor dopants [27] creating holes, such that SWCNT networks behave as *p*-type semiconductors.

In this paper, we consider sensing films consisting of disordered networks of SWCNT, for this kind of material it was shown that the conduction is due to the intra tube transport, which has a metallic behavior, and on the inter-tube transport. In particular, the conduction across the junctions between two nanotubes can be described [28,29,30,31] by fluctuation assisted tunneling across the inter-tube barriers. This last contribution usually dominates, whereas the first mechanism becomes important only at high temperatures when the tunneling probability becomes very large. Therefore, the following equation describes the conduction within the network [28]:

(1) $$\sigma^{-1}\left(T\right)=\rho \left(T\right)=B\exp\left(\frac{T_{1}}{T_{0}+T}\right)+A\exp\left(-\frac{T_{m}}{T}\right)$$

where σ is the film conductivity, ρ its resistivity, A and B are coefficients that can be considered constant with respect to temperature, $T_{m}$ is a characteristic temperature defining the metallic behavior, whereas $T_{1}$ and $T_{0}$ can be found from the following equations:

(2) $$T_{1}=\alpha \epsilon_{0}\;\frac{8A}{w}{\left(\frac{U}{q\;}\right)}^{2}=\beta^{2}U^{2}$$

(3) $$T_{0}=T_{1}\frac{h}{\pi^{2}w}\frac{1}{\sqrt{2mU}}$$

In Equations (2) and (3) *A* and w are the area and the width of the inter-tube junction, *h* is the Planck constant, $\epsilon_{0}\;$ is the vacuum permittivity, q is the electron charge, *m* is the free carrier effective mass, *α* is a constant depending on the molecule, and finally, *U* is the potential barrier height at the inter-tube junction. The value of $\;T_{0}$ was experimentally measured in SWCNT networks and usually found lower than 50 K (see Reference [32]). In the rightmost term of Equation (2) we define the parameter $\beta =\frac{1}{q}\sqrt{\frac{\alpha \epsilon_{0}8A}{w}}$, which can be considered a constant value.

Therefore, from now on in the model derivation, we will consider that the temperature is larger than 300 K and neglect $T_{0}$ in Equation (1). Moreover, since, as discussed in the introduction, we are interested in the behavior of low power sensors, we assume to operate at moderate temperature, and therefore, we assume the first term in Equation (1) being the most important. Therefore, from now on, the film conductance, *G*, will be described as follows:

(4) $$G=G_{0}\exp\left(-\frac{T_{1}}{T}\;\right)$$

where $G_{0}$ is a parameter depending on the film geometry and on parameter *B* in Equation (1), and as such, it is considered independent of temperature.

To obtain a complete model of a NO_2_ sensor, based on SWCNT networks, it is needed to relate the tunneling probability to the adsorbed gas surface density and to the target gas concentration.

To this aim, we consider that the sensors are used in air, therefore, in the presence of oxygen. Since there are many research studies showing that both oxygen and NO_2_ can be adsorbed on SWCNTs [33,34,35], we consider the following surface reactions [36,37]:

(5) $$\frac{1}{2}O_{2_{gas}}+S\begin{array}{c} \overset{k_{O}}{\to }\\ \underset{k_{iO}}{\leftarrow } \end{array}\left(O^{-}—S\right)$$

and:

(6) $$NO_{2}{}_{gas}+S\begin{array}{c} \overset{k_{NO2}}{\to }\\ \underset{k_{iNO2}}{\leftarrow } \end{array}\left(NO_{2}^{-}—S\right)$$

where *S* is an adsorption site whereas *k*_Y_ and *k_i_*_Y_ indicate the adsorption/desorption rate constants for the species *Y*. Moreover, we assume for all the reaction rate constants an Arrhenius form of the type:

(7) $$k_{Y}=k_{0Y}\exp\left(-\frac{\lambda_{Y}}{T}\right);\;\;k_{iY}=k_{0iY}\exp\left(-\frac{\lambda_{iY}}{T}\right)$$

From now on, the possible weak dependences on the temperature of all the pre-exponential terms, $k_{0Y}$ and $k_{0iY}$ are neglected.

In this paper, it was considered that the effect of the Au and TiO_2_ nanoparticles decoration is described by the selection of appropriate reaction kinetics for adsorption reactions (5) and (6). In fact, it is well known that these materials act as catalysts and favor the adsorption of the target gas. For example, it was shown that SWCNT decorated with both TiO_2_ and Au become more active at a lower temperature with respect to the pristine material [3].

In the subsequent modeling we use the following notation:

$$\left[O^{-}—S\right]=N_{0}\;\mathrm{and}\;\left[\;NO_{2}^{-}—S\right]=N_{NO2}$$

where [*Y*] is the surface density of the adsorbed specie *Y*. Therefore, the total density of the negative adsorbed charge is *N*s = $N_{s}$ = $N_{o}$ + $N_{NO2}$.

The kinetics of the two surface reactions can be described by the following differential equations:

(8) $$\frac{dN_{O}}{dt}=k_{O}{\left({\left[O_{2}\right]}_{gas}\right)}^{\frac{1}{2}}\left(\left[S\right]-N_{O}-N_{NO2}\right)-k_{iO}N_{O}$$

(9) $$\frac{dN_{NO2}}{dt}=k_{NO2}{\left[NO_{2}\right]}_{gas}\left(\left[S\right]-N_{O}-N_{NO2}\right)-k_{iNO2}N_{NO2}$$

where [*Z*]*_gas_* denotes the concentration of the gas *Z*. Equations (8) and (9) describe two first order reactions considering ionization as the rate limiting step. Notice that in Equations (8) and (9) a competitive adsorption was considered, therefore, the same adsorption sites were assumed as active for the two considered gases. If, instead, oxygen adsorption and NO_2_ adsorption occur at different adsorption sites ($S_{O}$ and $S_{NO2}$), the two equations decouple and become simply:

(10) $$\frac{dN_{O}}{dt}=k_{O}{\left({\left[O_{2}\right]}_{gas}\right)}^{\frac{1}{2}}\left(\left[S_{O}\right]-N_{O}\right)-k_{iO}N_{O}$$

(11) $$\frac{dN_{NO2}}{dt}=k_{NO2}{\left[NO_{2}\right]}_{gas}\left(\left[S_{NO2}\right]-N_{NO2}\right)-k_{iNO2}N_{NO2}$$

In this paper we assume that the height of the potential barrier, *U*, at inter-tube junction decreases linearly as a function of the adsorbed charge density, therefore, we assume:

(12) $$U=U_{0}-\gamma N_{s}=U_{0}-\gamma \left(N_{O}+N_{NO2}\right)$$

Under this hypothesis, the probability of tunneling is increased by the presence of the two oxidizing adsorbates, so it can be seen that $T_{1}$ varies as a function of $N_{s}$ according to the following equation:

(13) $$T_{1}=\beta^{2}{\left(U_{0}-\gamma N_{s}\right)}^{2}=\beta^{2}\left(U_{0}^{2}-2U_{0}\gamma N_{S}+\gamma^{2}N_{s}^{2}\right)=T_{10}-2\sqrt{T_{10}}\;\beta \gamma N_{S}^{}+\beta^{2}\gamma^{2}N_{S}^{2}$$

where $T_{10}$ represents the value of $T_{1}$ in the absence of any adsorbate.

From now on we define the scaled density of adsorbed species as follows:

(14) $$N_{s}^{\prime }=\gamma \beta N_{s};\;N_{O}^{\prime }=\gamma \beta N_{O};\;\;N_{NO2}^{\prime }=\gamma \beta N_{NO2}$$

With these positions we can rearrange Equations (8) and (9) as follows:

(15) $$\frac{dN_{O}^{\prime }}{dt}=k_{O}{\left({\left[O_{2}\right]}_{gas}\right)}^{\frac{1}{2}}\left({\left[S\right]}^{\prime }-N_{O}^{\prime }-N_{NO2}^{\prime }\right)-k_{iO}N_{O}^{\prime }$$

(16) $$\frac{dN_{NO2}^{\prime }}{dt}=k_{NO2}{\left[NO_{2}\right]}_{gas}\left({\left[S\right]}^{\prime }-N_{O}^{\prime }-N_{NO2}^{\prime }\right)-k_{iNO2}N_{NO2}^{\prime }$$

where [*S*]^′^ = γβ[*S*].

The combination of Equations (14)–(16) with Equation (4) can describe the response of the sensor in terms of conductance as a function of temperature and of oxygen and NO_2_ gas concentration. At constant temperature, being the model based on linear differential equations with coefficients that in this case are constant (reaction rates are constant), it can be used to derive an analytical description of the dynamic sensor response also in the presence of variable gas concentration. On the other hand, when considering a variable temperature, the coefficients of the differential equations are time-variant, and the analytical analysis becomes impractical. In this case, a numerical solution is possible. In both cases, before using the model to predict the sensor behavior analytically or numerically, it is necessary to estimate the model parameters, which are five both for Equations (15) and (16), and two for Equation (4).

## 4. Model Calibration: Parameter Estimation Procedure

The developed model was numerically implemented in Matlab, trying to minimize the number of parameters estimated and used to fit the experimental data. The differential equations were numerically solved and a non-linear least square fitting (lsqnonlin) was used to fit the experimental data and to find the unknown parameters of the model. The sensor temperature was measured by the embedded Pt-sensor and used as an input to the model, as well as the gas concentration, which is derived from the flowmeter settings.

The parameter $G_{0}$ was found with an independent methodology described in Reference [20], based on tests implying fast temperature transients. Since it is expected that the *N*_S_ dynamics is slower with respect to the thermal one, at the very beginning of the transients, *N*_S_, and therefore, *T*_1_, can be considered constant [20], therefore:

(17) $$\log\left(G_{0}\right)=\frac{T\left(t_{1}\right)\log\left(G\left(t_{1}\right)\right)-T\left(t_{2}\right)\log\left(G\left(t-2\right)\right)}{T\left(t_{1}\right)-T\left(t_{2}\right)}$$

where $t_{1}$ and $t_{2}$ are two instants in the first part of the transient, chosen to ensure that $T\left(t_{1}\right)\;\neq \;T\left(t_{2}\right)$ and that $N_{s}\left(t_{1}\right)\approx N_{s}\left(t_{2}\right)$. This is accomplished choosing t_1_ and t_2_ such that $t_{2}-t_{1}$ << τ_chemical,_ being τ_chemical_ a coarse estimation of the time constant of the fastest chemical reaction, derived from experimental data.

At first, the parameters used in Equations (15) and (16), ($k_{0O},\;\lambda_{O},\;k_{\mathrm{iO}},\;\lambda_{\mathrm{iO}},\;{\left[S\right]}^{\prime },\;and\;T_{10}$) were found by using measurements performed in pure dry synthetic air (no NO_2_ in the mixture, therefore, only reaction (5) involved).

Finally, the parameters of Equation (16) ($k_{0\mathrm{NO}2},\;\lambda_{\mathrm{NO}2},\;k_{\mathrm{iNO}2},\;\lambda_{\mathrm{iNO}2}\;$) were found using measurements in air and NO_2_ mixtures with different concentrations, using the parameters estimated in the air to numerically integrate Equation (15).

In particular, the fitting was obtained considering pulsed heating as proposed in this paper. Therefore, the measurements used to tune the model were obtained using the temperature profiles as those shown in the following figures. Where the desired temperature profile set by the temperature controller is a pulse train with period *t*_TEMP_ (in the range 1–2 min) and a small duty cycle.

**Figure 5:**
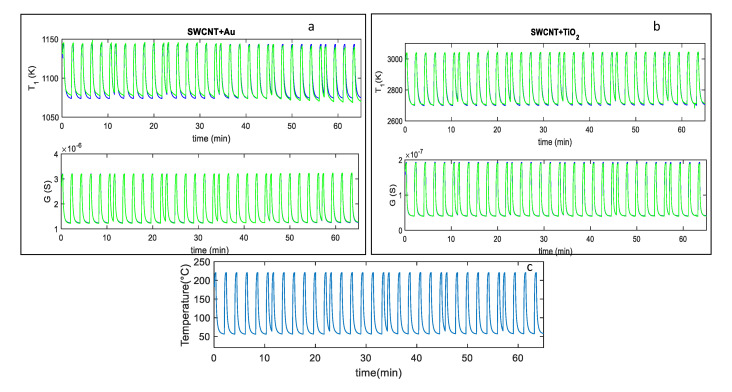
(**a**,**b**): Blue line-fitting results obtained after parameter estimation for the SWCNT-Au decorated samples (**a**), and the SWCNT-TiO_2_ decorated samples (**b**); green lines-measured values; the top plot reports the behavior of the parameter $T_{1},\;$whereas the bottom one reports the sensor response *G* vs. time. The reported results refer to measurements performed in dry synthetic air for approximately 65 min with the temperature profile shown in (**c**). (**c**) measured temperature profile, obtained by repeatedly heating at 220 °C for approximately 0.3 min, and at 60 °C for 1.6 min, respectively (total temperature pulse period *t*_p_ ≈ 2 min).

**Figure 6:**
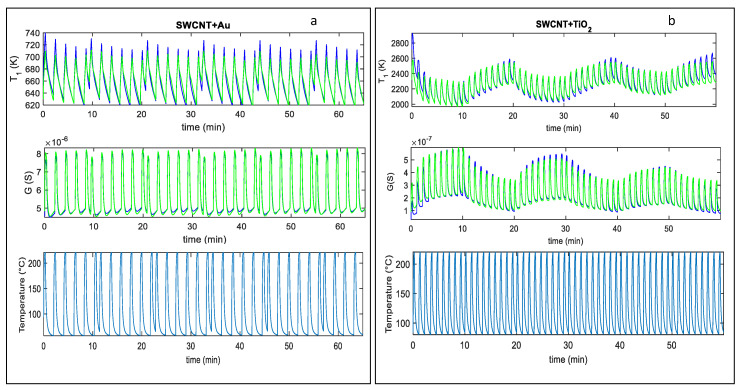
Fitting results obtained after parameter estimation for $T_{1}$ and *G* vs. time. (**a**), top two plots: Blue line-fitting results for the SWCNT-Au decorated sample, green lines-measured value; measurements obtained in a constant flow (200 mL/min) of air and 25 ppm NO_2_. (**a**), lower plot: Measured temperature profile, obtained by repeatedly heating at 220 °C for approximately 0.3 min, and at 60 °C for 1.6 min, respectively (total temperature pulse period *t*_p_ ≈ 2 min). (**b**), top two plots: Blue line-fitting results obtained for the SWCNT-TiO_2_ decorated sample, green lines-measured value; measurements obtained at constant flow (200 mL/min) with the following protocol: 10 min: air + 37 ppm NO_2_; 10 min: air; 10 min: air + 25 ppm NO_2_; 10 min: air; 10 min: air + 12 ppm NO_2_; 10 min: air. (**b**), lower plot: Measured temperature profile, obtained by repeatedly heating at 220 °C for approximately 0.2 min, and at 60 °C for 0.8 min, respectively (total temperature pulse period *t*_p_ ≈ 1 min).

In Figure 5a,b, an example of the fitting results obtained after parameter estimation for the two analyzed sensors, by using measurements in pure dry synthetic air are shown, whereas in Figure 6a,b the responses to mixtures of air and NO_2_ are shown. Notice that different measurements were used for parameter estimations leading to similar estimated values; this procedure was adopted to test the result consistency. Moreover, model verification was performed on additional measurements. The results, shown in Figure 6, concern two examples of model calibration obtained with two different profiles.

In particular, in Figure 6a, the NO_2_ concentration was kept constant (25 ppm) during the measurements, whereas in Figure 6b, three phases of different NO_2_ mixtures were considered (37 ppm, 25 ppm, 12 ppm), followed by recovery phases in synthetic air (as described in the figure captions). In all these figures, the estimated time behavior of the quantity $T_{1}$ and the measured temperature profile is also shown.

**Figure 7:**
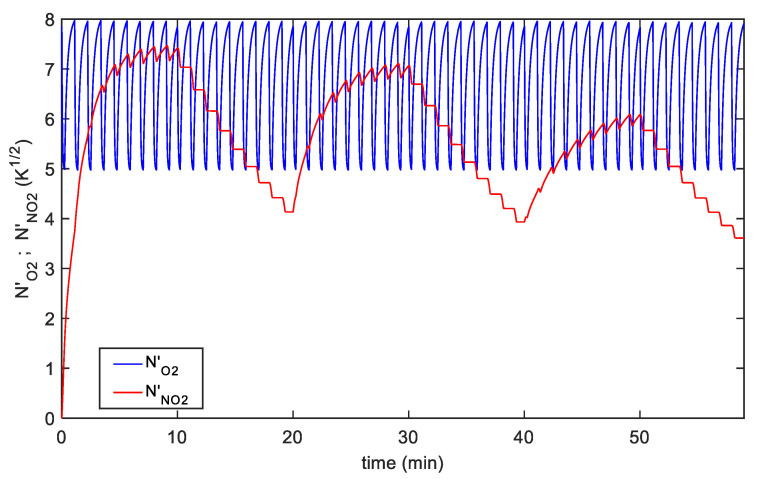
Time behavior of the normalized adsorbed species surface concentration: Red line-$N_{NO2}^{\prime }$, blue line-$N_{O2}^{\prime }$, the plot refers to the fitting results shown in Figure 6b.

The proposed model observes the behavior of the different kinetics of the two chemisorption reactions producing the sensors response, notice that these quantities cannot be recovered directly from the observation of the sensor output unless modeling is used. An example of the adsorbed species transient behavior can be seen in Figure 7. In this figure, the time behaviors of the normalized adsorbed species surface concentrations ($N_{\mathrm{NO}2}^{\prime },\;N_{O}^{\prime }$) relative to the fitting in Figure 6b for the TiO_2_ decorated SWCNT-based sensor are shown. In general, for both sensors, the simulated behaviors of the adsorbed NO_2_ and O_2_, show that the NO_2_ adsorption is favored at low temperatures, but that the adsorption reaction becomes very slow, so that it becomes difficult to exploit low temperature sensing in practical applications. On the other hand, the use of fast higher temperature pulses speeds up the overall dynamics and obtain remarkable sensor responses.

## 5. Simulation Results

The developed model, together with the estimated parameters, simulate the behavior of the sensors in many different conditions. In particular, the simulation explores the sensor performance obtained by pulsed temperature. The influence of the temperature profile characteristics on the sensor response was explored, varying all the parameters, i.e., the maximum *T*_high_ and the minimum *T*_low_ temperature, period, and duty cycle (i.e., duration of the high temperature pulse phase, *t*_h_).

It was considered to sample the sensor response after each heating pulse, therefore, a maximum period *t*_p_ of approximately 2 min was selected, the heating pulse duration (*t*_h_) and the maximum and minimum temperatures (*T*_high_ and *T*_low_) were varied.

The process of optimizing the pulse profile, under some constraints on the power consumption (maximum high temperature *T*_high_ with pulse duration *t*_h_) lead to the choice of *T*_high_ = 220 °C and max *t*_h_ = 0.3 min.

**Figure 8:**
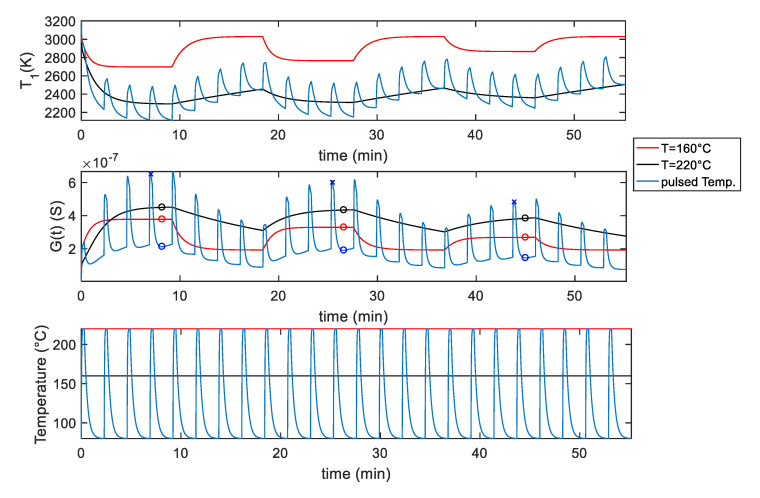
Simulation results for the *G* and *T*_1_ behavior for the SWCNT-TiO_2_ decorated sensor sample, assuming the temperature profiles shown in the bottom plot of the figure. The measurement gas profile is as follow: 9.2 min carrier gas + 37 ppm NO_2_, 9.2 min carrier gas, 9.2 min carrier gas + 25 ppm NO_2_, 9.2 min carrier gas, 9.2 min carrier gas + 12 ppm NO_2_, 9.2 min carrier gas, related temperature as per legend. For the markers, see Figure 9.

Figure 8 shows the simulation results for the SWCNT-TiO_2_ sensor obtained by using the selected ‘optimum’ pulsed temperature profile compared with the simulated response obtained using a constant temperature. In particular, the results obtained using the pulsed temperature between 220 °C and 80 °C and the results obtained using a constant temperature of 220 °C and the pulsed temperature average value (*T*_av_ = 160 °C) are reported. The temperature profiles used for simulations where generated by taking into account the thermal behavior of the sensors.

The simulation refers to the sensor response to gas concentration pulses, with values 37 ppm, 25 ppm and 12 ppm. Each gas phase (9.2 min) is followed by a pure air recovery phase with a duration of 9.2 min.

**Figure 9:**
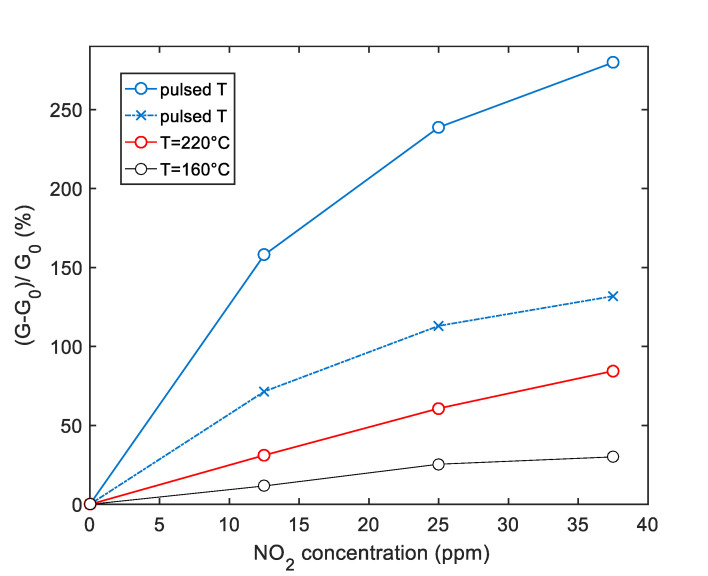
Simulated response of the sensor decorated with TiO_2_ to NO_2_ as a function of the NO_2_ concentration according to the selected temperature profile (shown in Figure 8). ×-blue markers: Results obtained by sampling the sensor response at the end of the last *T*_high_ pulse of each NO_2_ injection phase. O-blue markers: Results obtained by sampling the sensor response at the end of the last *T*_low_ pulse of each NO_2_ injection phase, as shown in Figure 8. Red and black lines are the responses obtained at the end of each $NO_{2}$ injection phase at a constant temperature as per legend.

According to the simulation results, the pulsed profile provides the possibility to highly enhance the sensor response. This is especially useful at very low gas concentrations, as highlighted in the results shown in Figure 9, for the TiO_2_ decorated sensor—where the sensors’ response (as a function of the NO_2_ concentration) is reported, and for each one of the simulated cases.

## 6. Experimental Results

In this section, the obtained experimental results will be presented and discussed, showing both that the proposed measurement technique can effectively enhance the performance of the tested SWCNT-based sensors, and also that the proposed model is sufficiently accurate so as to capture the most important features of the sensor behavior. Therefore, it can be conveniently used to design experiments and to optimize the measurement technique.

**Figure 10:**
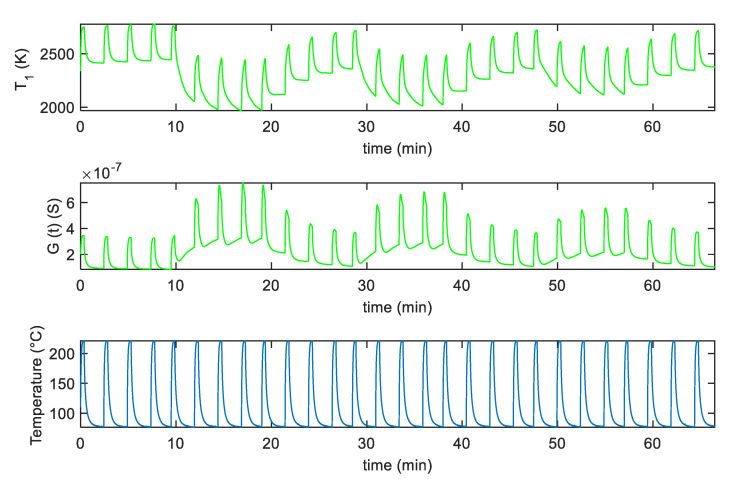
Measurement results for the *G* and *T*_1_ as a function of time for the SWCNT-TiO_2_ decorated sensor sample assuming the measurement profile as simulated in Figure 8 with an additional pure air phase preceding the NO_2_ injection.

**Figure 11:**
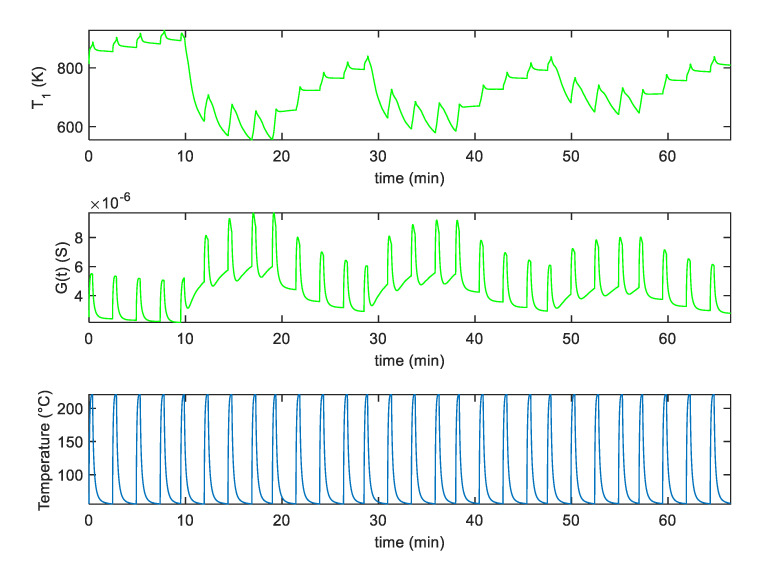
Measurement results for the *G* and *T*_1_ as a function of time for the SWCNT-Au decorated sensor sample assuming the measurement profile as simulated in Figure 8 with an additional pure air phase preceding the NO_2_ injection.

**Figure 12:**
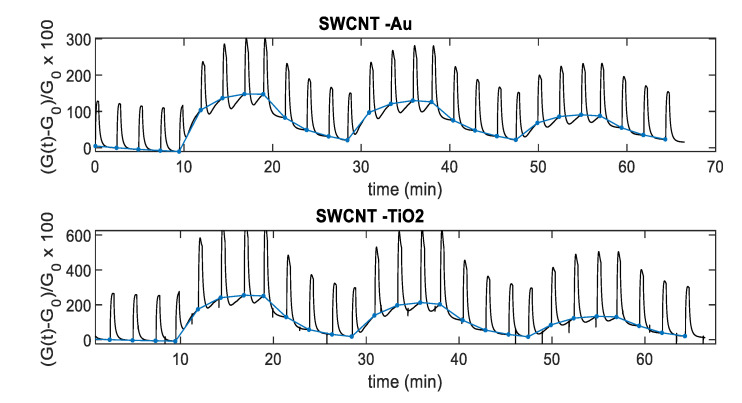
Black-line: Measurement results for the normalized conductance as a function of time for the two materials assuming the measurement profile as in Figure 11 and Figure 12. Blue-line: Responses sampled at the end of the low temperature (*T*_low_) phase.

**Figure 13:**
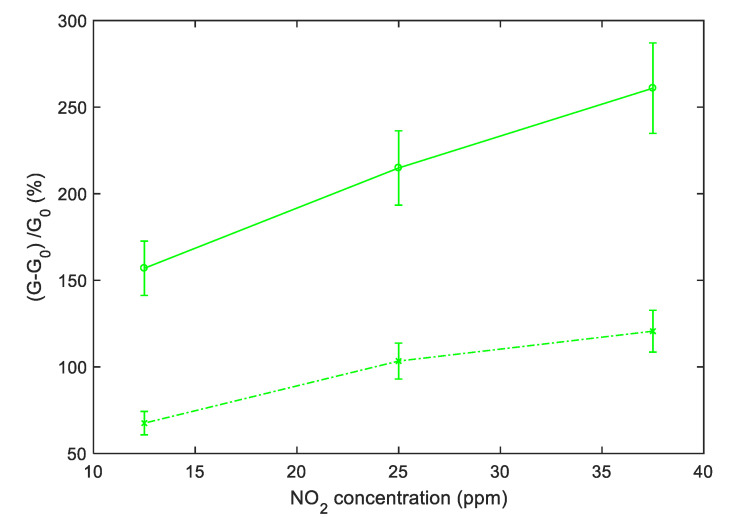
TiO_2_-decorated sensor response to NO_2_ as a function of the NO_2_ concentration using the temperature profile shown in Figure 8. Dashed line: Response sampled at the end of the last *T*_high_ pulse of each NO_2_ injection, solid line: Response sampled at the end of the last *T*_low_ pulse of each NO_2_ injection; the error bars refer to the estimated repeatability (approximately 10% of the response value).

As described in the previous section, the simulation analysis exploiting the derived model was used to find the ‘optimum temperature profile’. Accordingly, the optimum profile was used for measurements. Moreover, measurements with a constant temperature of *T*_high_ = 220 °C and *T*_av_ = 160 °C were conducted. The measurement results confirm the behavior predicted by the model, as it can be seen, for example, from data shown in Figure 10, Figure 11, Figure 12 and Figure 13.

In particular, Figure 10 shows the experimental results obtained with SWCNT-TiO_2_ sensor using the pulsed optimum temperature profile shown in Figure 8. In this figure, the obtained sensor response *G* and the assessed behavior of parameter $T_{1}$ are reported as a function of time. Figure 11 shows the same results for the sensor decorated with Au nanoparticles.

To obtain a convenient measurement output, the conductance obtained with pulsed temperature profiles can be synchronously sampled. As an example, Figure 12 shows the sensor responses that can be obtained by sampling the conductance at the end of the *T*_low_ phases. In particular, Figure 12 shows the sampling result for the relative sensor responses, where $G_{0}$ is the value of the conductance in air sampled at the end of the *T*_low_ phases. Note that the pulsed temperature limits the power consumption, obtaining very large responses and acceptable response and recovery times (5 min recovery time and 2 min response time 25 ppm NO_2_ for SWCNT-TiO_2_; 6 min recovery time and 2 min response time 25 ppm NO_2_ for SWCNT-Au).

**Figure 14:**
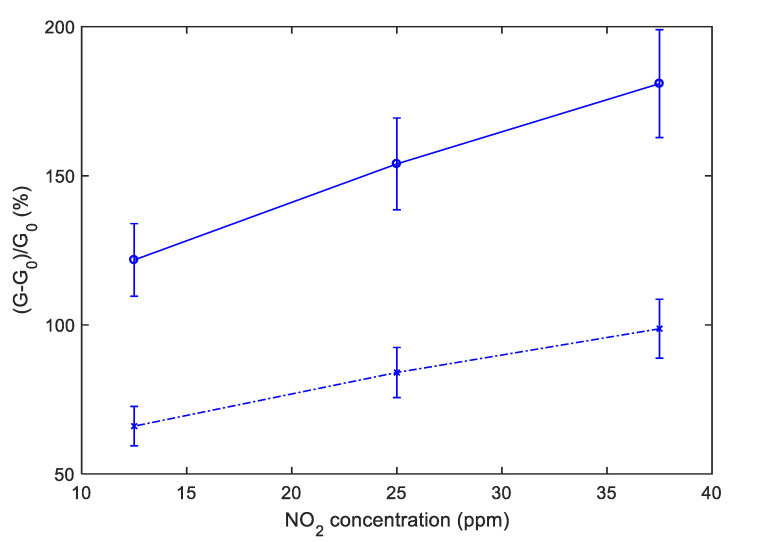
Au-decorated sensor response to NO_2_ as a function of the NO_2_ concentration using the measurement profile shown in Figure 8. Dashed line: Response sampled at the end of the last *T*_high_ pulse of each NO_2_ injection, solid line: Response sampled at the end of the last *T*_low_ pulse of each NO_2_ injection; the error bars refer to the estimated repeatability (approximately 10% of the response value).

Figure 13 and Figure 14 show, instead, the sensor responses as a function of NO_2_ concentration for the two materials, obtained both samplings, the conductance at the end of the *T*_high_ pulse and at the end of the *T*_low_ phase. It can be noticed that, similarly to what has been found for other gas-sensitive materials, and the best results are obtained by sampling the conductance at low temperature. The sensor responses plotted in Figure 13 and Figure 14 are obtained considering the value of the sensor conductance at the end of the gas exposure phase, and $G_{0}$ as the baseline value of conductance at the same temperature in air.

**Figure 15:**
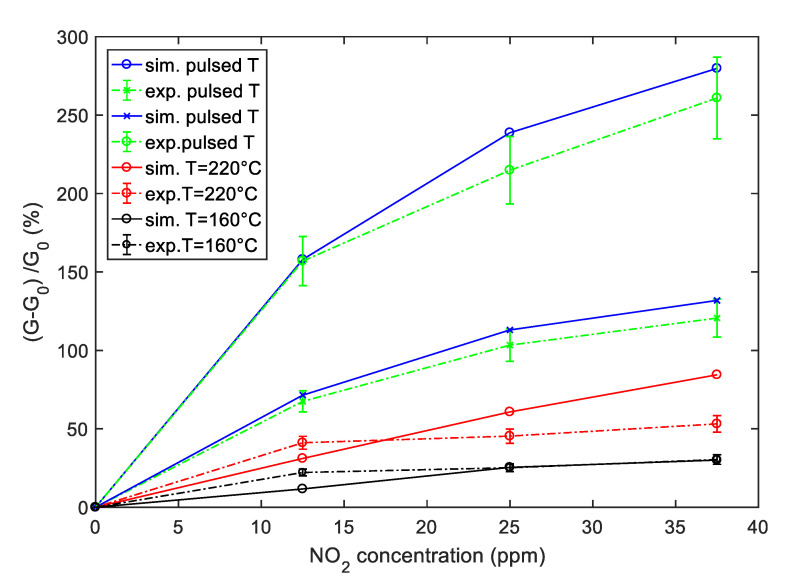
Simulation (solid lines) and experimental results (dashed lines) of the sensor response to NO_2_ as a function of the NO_2_ concentration for the SWCNT-TiO_2_ sample. Blue color—sensor response obtained with pulsed temperature measurement profile; red color—sensor response with a constant temperature at 220 °C, black color—sensor response with a constant temperature at 160 °C as in Figure 8; the error bars refer to the estimated repeatability (approximately 10% of the response value).

The comparison between the simulated sensor response obtained by using the derived model and the results obtained by experimental tests is summarized in Figure 15, where the responses are plotted as a function of NO_2_ concentration. The reported comparison refers to the SWCNT-TiO_2_ decorated sample, but the same analysis can be carried out as well, for the Au decorated sample.

It can be seen from Figure 15 that the simulation results are in good agreement with measurements in particular when the pulsed temperature mode is used; in fact, in this case, the estimated relative error is less than 10%. On the other hand, the deviation between predicted and experimental responses can be higher for measurements with a constant temperature. In any case, as predicted by the model, the pulsed temperature technique remarkably enhances the sensor response magnitude, with respect to constant working temperature, both using the peak temperature (*T*_high_) and the low temperature (*T*_low_) sampling. The enhancement is obtained both respect to measurements performed at a constant temperature T = *T*_high_ = 220 °C and T = *T*_av_ = 160 °C as in Figure 8.

The mean power consumption during the measurement has been estimated. In particular, assuming the selected optimum pulsed temperature profile as in Section 3 and Section 4 (i.e., *T*_high_ = 220 °C, *T*_low_ = 80 °C, *t*_p_ = 2 min, *t*_h_ = 0.3 min), by writing the pulse period *t*_p_ = *t*_h_ + *t*_l_, the estimated mean power consumption *P_m_* during a pulse period is:

(18) $$P_{m}=\frac{P_{h}\cdot t_{h}+P_{l}\cdot t_{l}}{t_{p}}\approx 470\;mW$$

where *P*_h_ and *P*_l_ are the power required for heating the sensor at 220 °C and at 80 °C, respectively, whereas the estimated *P*_m_ assuming a constant temperature of 220 °C is approximately 1.01 W.

## 7. Conclusions

In this paper, we have demonstrated the possibility of enhancing the sensitivity of SWCNT sensors by using pulsed temperature. Moreover, we developed a novel model, which predicts the conductance of SWCNT networks-based gas sensors working with variable gas concentration and temperature. This model is the first, in the authors’ knowledge, that provides the description of the main chemical and physical phenomena, which relate the conductance of the network to the gas concentration (NO_2_), and accounts for the influence of temperature variations. The model has allowed for choosing an ‘optimum’ temperature profile, replacing with simulations the experimental tuning, which is an extremely long procedure. Moreover, the developed model has given the possibility of further the knowledge on SWCNT sensor behavior, providing the description of quantities that determine the final sensor output, but that cannot be directly observed or measured, as, for instance, the transient behavior of the different adsorbed species. In this respect, it has been seen that the adsorption of NO_2_ is highly favored by low temperatures, but due to the very slow kinetics, the observed variation of conductance during tests with gas exposures phases of reasonable durations (tens of minutes) remains very low.

According to the investigations carried out, using the pulsed measurement profile, it is possible to remarkably enhance the sensor sensitivity to NO_2_, and particularly for a very low concentration, i.e., 12 ppm. Moreover, the results reveal that by sampling the sensor response at the end of low temperature *T*_low_ phase the sensors reach a maximum of the sensing capability.

As a final remark, it must be stressed that the profiles investigated through simulations and among which we selected the optimum profile, were those that could be actually obtained with the prototype sensors used in this work, which have a quite large thermal capacitance—therefore, the pulse train periods considered cannot be shorter than five times the thermal time constant. Nevertheless, by using smaller sensors with this technique, faster pulse trains could be taken into account.

From a power consumption point of view, the pulsed temperature measurement profile grants a power-saving of approximately 50% with respect to the constant temperature measurement profile which ensures an appreciable sensitivity performance (i.e., T = 220 °C selected for comparison).
